# Effect of docosahexaenoic acid as an anti-inflammatory for Caco-2 cells and modulating agent for gut microbiota in children with obesity (the DAMOCLE study)

**DOI:** 10.1007/s40618-024-02444-w

**Published:** 2024-08-26

**Authors:** C. Lammi, E. Ottaviano, G. Fiore, C. Bollati, L. d’Adduzio, M. Fanzaga, C. Ceccarani, S. Vizzuso, G. Zuccotti, E. Borghi, E. Verduci

**Affiliations:** 1 Department of Pharmaceutical Sciences, University of Milan, 20133 Milan, Italy; 2 Department of Health Sciences, University of Milan, 20142 Milan, Italy; 3 Department of Pediatrics, Vittore Buzzi Children’s Hospital, University of Milan, Via Lodovico Castelvetro 32, 20154 Milan, Italy; 4 Institute for Biomedical Technologies, CNR, Segrate, Italy; 5 Department of Biomedical and Clinical Sciences, University of Milan, 20157 Milan, Italy; 6 Metabolic Diseases Unit, Department of Paediatrics, Vittore Buzzi Children’s Hospital, University of Milan, 20157 Milan, Italy

**Keywords:** Docosahexaenoic acid, Gut microbiota, Childhood obesity, Caco-2 cells, Antioxidant effect, Anti-inflammatory activity

## Abstract

**Purpose:**

Docosahexaenoic acid (DHA) is a long-chain omega‐3 polyunsaturated fatty acid. We investigated the dual health ability of DHA to modulate gut microbiota in children with obesity and to exert anti-inflammatory activity on human intestinal Caco-2 cells.

**Methods:**

In a pilot study involving 18 obese children (8–14 years), participants received a daily DHA supplement (500 mg/day) and dietary intervention from baseline (T0) to 4 months (T1), followed by dietary intervention alone from 4 months (T1) to 8 months (T2). Fecal samples, anthropometry, biochemicals and dietary assessment were collected at each timepoint. At preclinical level, we evaluated DHA’s antioxidant and anti-inflammatory effects on Caco-2 cells stimulated with Hydrogen peroxide (H_2_O_2_) and Lipopolysaccharides (LPS), by measuring also Inducible nitric oxide synthase (iNOS) levels and cytokines, respectively.

**Results:**

Ten children were included in final analysis. No major changes were observed for anthropometric and biochemical parameters, and participants showed a low dietary compliance at T1 and T2. DHA supplementation restored the Firmicutes/Bacteroidetes ratio that was conserved also after the DHA discontinuation at T2. DHA supplementation drove a depletion in *Ruminococcaceae* and *Dialisteraceae,* and enrichment in *Bacteroidaceae*, *Oscillospiraceae,* and *Akkermansiaceae*. At genus level, *Allisonella* was the most decreased by DHA supplementation. In Caco-2 cells, DHA decreased H_2_O_2_-induced reactive oxygen species (ROS) and nitric oxide (NO) production via iNOS pathway modulation. Additionally, DHA modulated proinflammatory (IL-1β, IL-6, IFN-γ, TNF-α) and anti-inflammatory (IL-10) cytokine production in LPS-stimulated Caco-2 cells.

**Conclusion:**

An improvement in gut dysbiosis of children with obesity seems to be triggered by DHA and to continue after discontinuation. The ability to modulate gut microbiota, matches also with an anti-inflammatory effect of DHA on Caco-2 cells.

**Graphical abstract:**

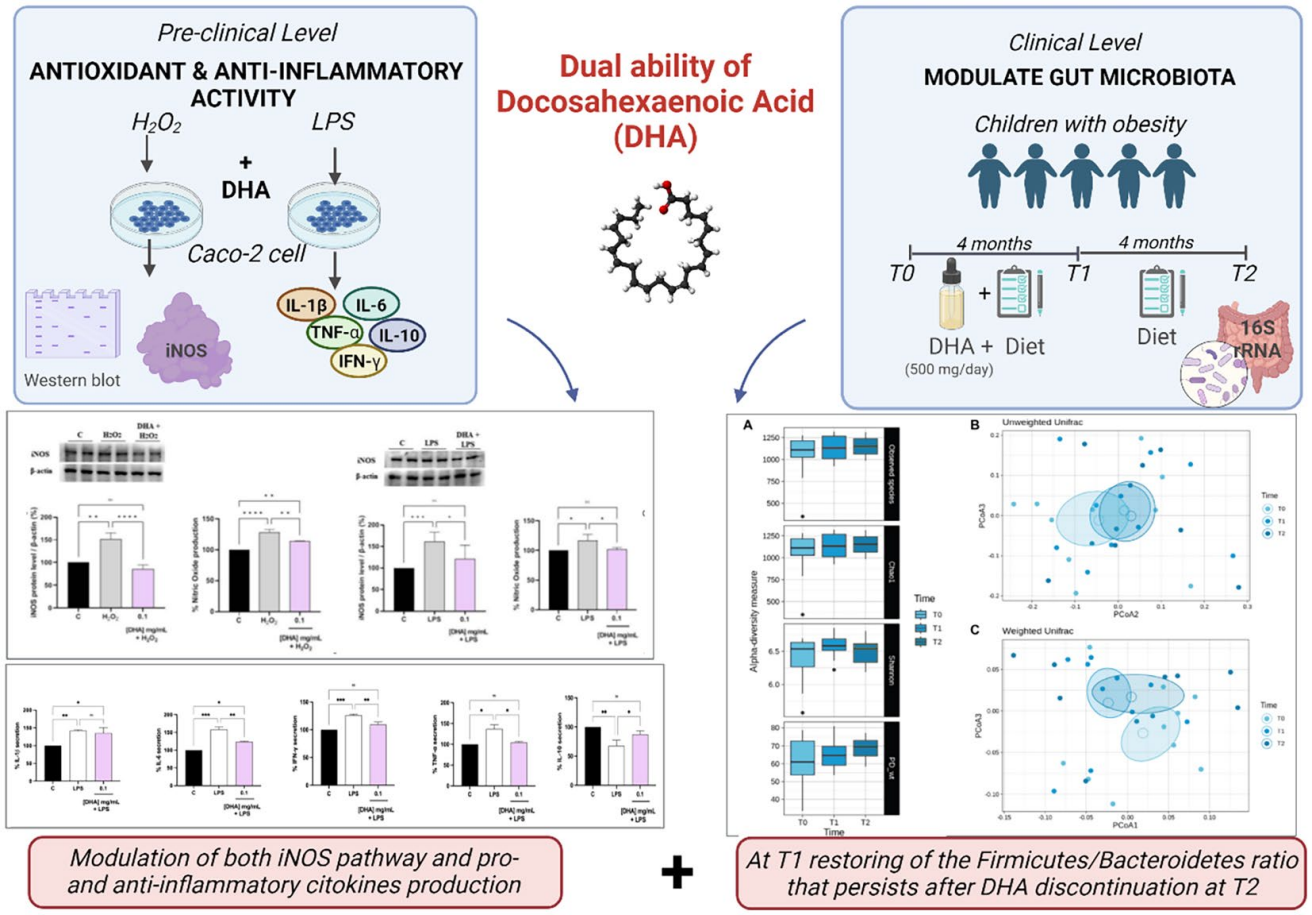

**Supplementary Information:**

The online version contains supplementary material available at 10.1007/s40618-024-02444-w.

## Introduction

Polyunsaturated fatty acids (PUFAs) are principally divided into the omega-6 and the omega-3 families, essential fatty acids for humans. Linoleic acid (LA) and arachidonic acid (AA) are omega-6 fatty acids and α-linolenic acid (ALA), eicosapentaenoic acid (20:5*n*‐3, EPA) and docosahexaenoic acid (22:6*n*‐3, DHA) are omega‐3 long‐chain PUFA (n*‐*3 PUFA) [1]. Omega-3 fatty acids are generally considered anti-inflammatory, and different mechanisms are described [2]. Incorporation of omega-3 fatty acids in the cell membrane lowers the content of AA, and thereby the substrate for synthesis of AA-derived eicosanoids, which are generally considered more pro-inflammatory than eicosanoids derived from EPA [2].

Notably, DHA is critical for all life stages from the need for fetal development, the prevention of preterm birth, and the prevention of cardiovascular disease to the improvements in the cognitive function and the eye health from early life to adulthood and elderly. The recommended intake of DHA depends on many factors, such as regions, age groups, and genders. DHA supplements are mainly from fish, krill, and algal oils, and the bioavailability of DHA depends on its chemical structure and matrix. For example, DHA in phospholipid and triglyceride forms are more readily absorbed by the body than that in ethyl ester form. In addition, dietary lipids in meals and emulsification of DHA oil can increase the bioavailability of DHA.

There are many studies in the literature showing the health benefits of DHA. These benefits might be related to different mechanisms of actions such as the anti-inflammatory activity at intestinal level and the modulation of gut microbiota by DHA [3]. Although the data are still limited to date, some preclinical studies have shown that DHA possesses prebiotic effects. n‐3 PUFAs indirectly or directly modulate the gut microbiota, which leads to the reduction of pro‐inflammatory levels and the increase in short‐chain fatty acids (SCFAs) [4]. Some animal studies investigated the prebiotic role of DHA supplementation in high-fat diet mice [5–10]. After high-DHA tuna oil supplementation, a dramatic reduction in obesity-promoting bacteria and a positive modification in gut microbiota composition were observed [5, 6, 9]. DHA-supplemented mice showed more abundant propionic/butyric acid-producing bacteria and less-abundant lipopolysaccharide-correlated species [7, 8]. It has been documented that transferring microbiota from fish-oil-fed mice reduced high-fat-diet-induced weight gain, insulin resistance (IR) and inflammation [10]. Similarly, the transplantation of DHA/EPA-modulated microbiota in db/db mice mimicked the ameliorative effect of DHA/EPA on glucose homeostasis, and brought similar changes in gut metabolites [7].

At clinical level, evidence of DHA prebiotic role is still scanty. A randomized, open‐label, cross‐over trial providing 4 g/day of the combination of DHA and EPA (or 2 g DHA) in triglyceride or ethyl ester form to healthy adults for 8 weeks reported that both DHA forms significantly increased the abundance of several beneficial genera [11], such as *Bifidobacterium, Lactobacillus* and *Roseburia*, with the latter being regarded as one of the main producers of butyrate in the gut. Butyrate is a SCFA deemed as important nutrient for the colonic mucosa, which in turn modulates gene expression, inflammation, differentiation, and apoptosis in host cells [4].

There is consolidated evidence that obesity alters gut microbiota composition and reduces gut microbial diversity among the adult population [12, 13]. The same is under investigation among children and adolescents with obesity [14–17]. Specifically, dietary interventions are being studied that can restore the gut microbiota and its metabolic activity to levels similar to those of normal-weight children [18–20]. We previously reported an increase Firmicutes/Bacteroidetes (F/B) ratio in a group of children with obesity compared to normal weight controls [21], with differences in relative abundance of some core microbial species, including *Akkermansia muciniphyla*, *Faecalibacterium prausnitzii*, Bacteroides/Prevotella group, *Candida* spp., and *Saccharomyces* spp. [22].

The effect of DHA might be, at least in part, accountable for other health benefits in children with obesity, including the anti‐inflammatory properties.

Accordingly, blood concentrations of n-3 long-chain PUFA and especially of DHA are reduced in plasma and red blood cell phospholipids of children with obesity [23, 24]. The lower n-3 long-chain PUFA status in children with obesity is consistent with suboptimal dietary intakes of omega-3 and DHA observed [23, 24]. DHA is associated with multiple health benefits, specifically is precursor of specialized pro-resolving mediators (SPMs), such as resolvins, protectins, and maresins that modulate the inflammatory response by decreasing cytokine production and promoting the resolution of inflammation [25, 26]. Since obesity is a systemic low-grade inflammation associated with increased plasma levels of inflammatory markers including C-reactive Protein (CRP) and IL-6 [27, 28], the potential role of DHA as an anti-inflammatory agent has to be investigated in children with obesity. Meanwhile, evidence of anti-inflammatory properties of DHA on intestinal inflammation is emerging in vitro cell line models [29–31].

Based on these considerations, this pilot study aims to investigate the dual health ability of DHA to modulate the gut microbiota in children with obesity and to exert anti-inflammatory and antioxidant activities on human intestinal Caco-2 cells. Firstly, we evaluated the effect of DHA supplementation, combined with interventions aimed at improving diet and lifestyle, on gut microbiota composition in a group of children with obesity aged 8–14 years old.

After, we studied on human intestinal Caco-2 cells the ability of DHA to scavenge oxidative and inflammatory status. In addition, the effect of DHA in the activation of the inducible-nitric oxide synthase (iNOS) pathway was evaluated and in parallel, pro-inflammatory (IL-1β, IL-6, IFN-γ, and TNF-α) and anti-inflammatory (IL10) cytokines levels were measured on the same cellular system.

## Materials and methods

### Study design and participants

In this pilot study, 18 Caucasian children were enrolled at the Pediatric Department of San Paolo Hospital, Milan, Italy from January to July 2019. Eligibility criteria for participants were: age 8–14 years, diagnosis of obesity (defined as >  + 2 standard deviation score (SDS) on Body Mass Index (BMI) for-age according to World Health Organization (WHO) growth charts) [32], children living in Northern Italy born from Caucasian parents with minimum birth weight of 2500 g, gestational age 37–42 weeks and singleton birth. Children with secondary obesity, antibiotic or pre/pro/synbiotic treatment in the previous 3 months, neonatal disease, congenital malformations, affected by chronic or acute intestinal disease were excluded.

The study “Effect of Docosahexaenoic Acid supplementation on Microbiome in Obese ChiLdrEn (DAMOCLE Study)” was conducted according to the guidelines of the Declaration of Helsinki and approved by the Institutional Review Board of the hospital and registered on ClinicalTrials.gov (identifier: NCT04151758). Children’s parents gave their written consent for inclusion after being informed about the nature of the study. Data concerning mode of delivery and type of feeding were asked to all subjects during enrollment to verify whether patients fulfill the previously describes inclusion criteria. After enrollment, all children were evaluated at baseline (T0), at 4 months (T1), and at 8 months (T2). From T0 to T1 children were supplemented with DHA and received a targeted dietary intervention for obesity, whilst from T1 to T2 supplementation was discontinued and only dietary intervention was pursued. After enrollment, all participants underwent at T0, T1, and T2 a complete examination in the pediatric obesity outpatient clinic, including a medical examination, dietary evaluation, anthropometric and biochemical assessment. Clinical examination was performed by a physician including Tanner Score evaluation, Bristol Stool Chart identification, and systolic (SBP) and diastolic blood pressure (DBP) (mmHg) assessment. Parents were asked to collect 24 h before medical examination fecal samples, store it at −20 °C and bring it to visit the following day. Stool samples were collected at the hospital and stored at −80 °C until the microbiota analysis.

### Study intervention

After baseline evaluation, children were instructed to take a dose of 2 ml/day of DHA oil product of microalgal origin (*Schizochytrium* sp.), for a total of 500 mg/day of DHA. To ensure the 4 months supplementation, a total of 8 bottles of the product, each containing 30 ml of oil, were provided to families.

Together with supplementation children started a behavior (promotion of physical activity) and dietary treatment according to Italian dietary guidelines for childhood obesity [33, 34]. A nutritionist performed counselling based on the principles of the Mediterranean diet, providing advice on healthy eating to all the enrolled patients and caregivers. After, each participant was provided with a balanced and isocaloric dietary intervention according to sex and age to meet the National Recommended Energy and Nutrient Intake Levels, as per indication of the Italian Society of Pediatrics [35]. To assess supplementation and dietary compliance, families received telephone calls during the study period, specifically two telephone calls between T0 and T1 and other two between T1 and T2.

### Anthropometric and biochemical measurements

At each timepoint (T0, T1, T2) anthropometric parameters and body composition assessments were carried out for each patient. Body weight and height were measured using a mechanical column scale with an integrated measuring rod. BMI and related SDS were calculated from the ratio of weight to height squared (kg/m2) using the WHO reference growth charts [32]. Waist circumferences (WC) and tricipital skinfold thickness (TSF) were measured with a measuring tape (Seca 201) and a caliper (Holtain 610) respectively, and their z scores, WCz and TSFz, were calculated accordingly [36, 37]. Waist-to-height-ratio (WHtR) was calculated as the ratio between WC and height in cm, and a WHtR ≥ 0.55 was considered as an abdominal adiposity marker [38]. Body composition assessment was performed using Air displacement plethysmography through a BOD POD® device (COSMED, Life Measurements, Inc, Concord, CA) to obtain Fat Mass (FM) and Fat Free Mass (FFM) in % and kg, and the related FM index (FMI) and FFM index (FFMI) were calculated.

Fasting blood samples were collected from each participant at each visit. To evaluate glucose metabolism insulin (mIU/ml), glucose (mg/dL), and glycated hemoglobin (mmol/mol) were measured and related Homeostasis Model Assessment of Insulin resistance (HOMA-IR) [Inulin mIU x Glucose (mmol/L)/22.5] [39], Quantitative Insulin-Sensitivity Check Index (QUICKI) [1/logInsulin(mU/mL) + logGlucose(mg/dL)] [40], and McAuley index [e^(2.63—0.28 × LnInsulin(mU/L)—0.31 × LnTriglycerides(mmol/L)][41] were calculated as previously reported. Total cholesterol (mg/dL), Low-density lipoproteins cholesterol (LDL-C) (mg/dL), High-density lipoproteins cholesterol (HDL-C) (mg/dL), triglycerides (TG) (mg/dL), aspartate aminotransaminase (AST), alanine aminotransferase (ALT), gamma-glutamyl transferase (GGT) and erythrocyte sedimentation rate (ESR) were also measured. Lastly Triglyceride–Glucose index (TyG) [Ln[Tryglycerides(mg/dL)xGlucose(mg/dL)]/2], and Visceral Adiposity index (VAI) were assessed according to previous publications [42–44].

### Dietary assessment and compliance

Dietary assessment was performed collecting at each visit a 3-day weighed food record. Parents were advised on how to record all food and beverages consumed during 2 weekdays and 1 weekend day. Quantification and analysis of the energy and dietary intakes at T0, T1, and T2 were performed with an ad hoc nutritional software (MètaDieta®, MetaDieta srl). Information on energy intake (kcal/day), carbohydrates (CHO%En), sugars (%En), glycemic index (GI), fibers (g/day), lipids (%En), saturated fatty acids (SFA%En), monounsaturated fatty acids (MUFA%En), PUFAs (PUFA%En), and proteins (total g/day and g/kg/day) were retrieved. To assess adherence to the Mediterranean dietary pattern the KIDMED questionnaire was administrated. The KIDMED index ranges from 0 to 12 points and is based on a 16-question test that can be self-administered. The final score of the questionnaire were classified as follows: (a) 8 or more, optimal adherence to Mediterranean diet; (b) 4–7, improvement needed to adjust intake to a Mediterranean-like dietary patterns; (c) 3 or less, very low diet quality [45, 46].

### Fecal DNA extraction and 16S rRNA sequencing

Bacterial DNA was extracted from fecal samples using the QIAamp Powerfecal DNA kit (Qiagen. Germany) according to the manufacturer’s instructions. DNA concentration was quantified by Qubit® (Thermo Fisher Scientific. Waltham. WA), and stored until use at − 20 °C. The sequencing of the V3–V4 hypervariable regions of the bacterial 16S rRNA gene was performed by a service offered by Macrogen (Seoul. Republic of Korea), according to the Illumina 16S Metagenomic Sequencing Library Preparation (Illumina. San Diego. CA. USA).

### Bioinformatic analysis

Amplicon sequence variants (ASVs) were identified from 16S paired‐end sequencing using the Divisive Amplicon Denoising Algorithm (DADA2. version 1.18.0) pipeline, including filtering and trimming of the reads (version 1.16.0) [47]. Reads per sample were trimmed to 25,000 reads in order to compensate for the sequencing unevenness of the samples and to provide a consistent minimum amount for the downstream analysis. carried out through the “phyloseq” package (version 1.34.0) [48]. Alpha‐diversity evaluation was performed according to several microbial diversity metrics (i.e., chao1, Shannon Index, Observed species); the Faith’s phylogenetic tree diversity metric (“PD whole tree”) was elaborated through the “btools” package. Beta‐diversity analysis was conducted using unweighted Unifrac metrics and the principal coordinates analysis (PCoA).

Taxonomy was assigned to the ASVs using the 8‐mer‐based classifier from the 11.5 release of the Ribosomal Database Project (RDP) database and using the Genome Taxonomy Database (GTDB) 16S rRNA database (release r207) (release r207) [49].

### Chemicals

Dulbecco’s modified Eagle’s medium (DMEM), L-glutamine, fetal bovine serum (FBS), phosphate buffered saline (PBS), penicillin/streptomycin, chemiluminescent reagent, and 24 or 96-well plates were purchased from Euroclone (Milan, Italy). MTT [3-(4,5-dimethylthiazol-2-yl)-2,5-diphenyltetrazolium bromide], DPPH (1,1-diphenyl-2-picrylhydrazyl), TPTZ (2,4,6-Tris(2-pyridyl)-s-triazine), Griess reagent, bovine serum albumin (BSA), Radioimmunoprecipitation assay (RIPA) buffer, the antibody against β-actin, fluorometric intracellular ROS kit and Malondialdehyde (MDA) assay kit were bought from Sigma-Aldrich (St. Louis, MO, USA). Phenylmethanesulfonyl fluoride (PMSF), Na-orthovanadate inhibitors, and the antibodies against rabbit Ig-horseradish peroxidase (HRP) and mouse Ig-HRP were purchased from Santa Cruz Biotechnology Inc. (Santa Cruz, CA, USA). The iNOS primary antibody came from Cell Signaling Technology (Danvers, MA, USA); the inhibitor cocktail Complete Midi from Roche (Basel, Swiss); Mini protean TGX pre-cast gel 7.5% and Mini nitrocellulose Transfer Packs from BioRad (Hercules, CA, USA).

### DPPH (2,2-diphenyl-1-picrylhydrazyl radical scavenging) assay

1,1-Diphenyl-2-picrylhydrazyl radical (DPPH) assay was performed to determine the antioxidant activity by standard method with a slight modification. Briefly, the DPPH solution (12.5 μM in methanol, 45 μL) was added to 15 μL of DHA samples at different concentrations (0.1–1.0 mg/mL) in a 96-well half area plate. The reaction for scavenging DPPH radicals was performed in the dark at room temperature and the absorbance was measured at 520 nm after 30 min incubation.

### FRAP assay

The ferric reducing ability of plasma (FRAP) assay evaluates the ability of a sample to reduce ferric ion (Fe^3+^) into ferrous ion (Fe^2+^). Thus, 10 µL of the sample (DHA 15X) were mixed with 140 µL of FRAP reagent. The FRAP reagent was prepared by mixing 1.3 mL of a 10 mM TPTZ (Sigma-Aldrich, Milan, Italy) solution in 40 mM HCl, 1.3 mL of 20 mM FeCl_3_ × 6H_2_O and 13 mL of 0.3 M acetate buffer (pH 3.6). The microplate was incubated for 30 min at 37 °C and the absorbance was read at 595 nm. Absorbances were recorded on a Synergy™ HT-multimode microplate reader.

### Cell culture

Caco-2 cells, obtained from INSERM (Paris, France), were routinely sub-cultured at 50% density and maintained at 37 °C in a 90% air/10% CO2 atmosphere in DMEM containing 25 mM of glucose, 3.7 g/L of NaHCO_3_, 4 mM of stable l-glutamine, 1% nonessential amino acids, 100 U/L of penicillin, and 100 μg/L of streptomycin (complete medium), supplemented with 10% heat-inactivated fetal bovine serum (FBS; Hyclone Laboratories, Logan, UT, USA).

### 3-(4,5-Dimethylthiazol-2-yl)-2,5-diphenyltetrazolium bromide (MTT) assay

A total of 3 × 10^4^ Caco-2 cells/well were seeded in 96-well plates and treated with DHA from 0.1 to 10.0 mg/mL, or vehicle, in complete growth media for 48 h at 37 °C under 5% CO_2_ atmosphere. Subsequently, the treatment solvent was aspirated and 100 µL/well of MTT filtered solution added. After 2 h of incubation at 37 °C under 5% CO2 atmosphere, 0.5 mg/mL solution was aspirated and 100 µL/well of the lysis buffer [8 mM HCl + 0.5% NP-40 in dimethyl sulfoxide (DMSO)] added. After 10 min of slow shaking, the absorbance at 575 nm was read on the Synergy H1 fluorescence plate reader (Biotek, Bad Friedrichshall, Germany).

### Fluorometric intracellular ROS assay

For cell preparation, 3 × 10^4^ Caco-2 cells/well were seeded on a black 96-well plate overnight in growth medium. The day after, the medium was removed and replaced with 50 μL/well of the Master Reaction Mix and the cells were incubated at 5% CO_2_, 37 °C for 1 h in the dark. Then, cells were treated with 5 μL of 11X DHA (to reach the final concentrations of 0.1 and 1.0 mg/mL) and incubated at 37 °C for 24 h in the dark. To induce ROS, cells were treated with 5 μL of H_2_O_2_ at a final concentration of 1.0 mM for 30 min at 37 °C in the dark and fluorescence signals (ex./ em. 490/525 nm) were recorded using a Synergy H1 microplate reader.

### Lipid peroxidation (MDA) assay

Caco-2 cells (2.5 × 10^5^ cells/well) were seeded in a 24 well plate and, the following day, they were treated with DHA (at the final concentrations of 0.1 and 1.0 mg/mL for 24 h at 37 °C under 5% CO_2_ atmosphere. After incubation, cells were stimulated with H_2_O_2_ 1 mM, or vehicle, for 30 min, than collected and homogenized in 150 µL ice-cold MDA lysis buffer containing 1.5 µL of Butylated hydroxytoluene (BHT) (100x). Samples were centrifuged at 13,000×*g* for 10 min, then they were filtered through a 0.2 µm filter to remove insoluble material. To form the MDA-thiobarbituric acid (TBA) adduct, 300 µL of the TBA solution were added into each vial containing samples and incubated at 95 °C for 60 min, then cooled to room temperature (RT) for 10 min in an ice bath. For analysis, 100 µL of each reaction mixture were pipetted into a 96 well plate and the absorbance was measured at 532 nm using the Synergy H1 fluorescent plate reader from Biotek.

### Nitric oxide level evaluation on Caco-2 cells

Caco-2 cells (1.5 × 10^5^/well) were seeded on 24-well plates. The next day, cells were treated for 24 h with DHA to reach the final concentrations of 0.1 mg/mL and incubated at 37 °C under a 5% CO_2_ atmosphere. After incubation, cells were stimulated with H_2_O_2_ (1.0 mM) or vehicle for 1 h, then the cell culture media were collected and centrifuged at 13,000×*g* for 15 min to remove insoluble material. NO determination was carried out by Griess test. Briefly, 1.0 g of Griess reagent powder was dissolved in 25.0 ml of distilled H_2_O and 50 μL of the solution were incubated with 50 μL of the culture supernatants for 15 min at RT in the dark. The absorbance was measured at 540 nm using the Synergy H1 fluorescent plate reader from Biotek.

### Cytokines quantification

Cytokines quantification was performed using human Quanttikine® ELISA kits (R&D Systems, Minneapolis, MN, USA), according to the manufacturer’s instructions. Briefly, the supernatants collected from treated and LPS-stimulated Caco-2 cells were centrifuged at 13,300×*g* for 10 min at 4 °C, then the pellet and insoluble material were discarded. For the experiments, 100 µL of samples were added to each well and the microplate was incubated for 2 h at RT. After the incubation, the solutions were discarded and each well washed 4 times with 300 µL of Wash Buffer solution, then 200 µL of Conjugate solution were added to each well and the microplate was incubated for 2 h at RT. After incubation, the solutions were discarded and each well washed 4 times with 300 µL of Wash Buffer solution; then 200 µL of Substrate Solution were added and the plate was incubated for 1–4 h at RT in the dark. The reactions were stopped with 50 µL of Stop Solution and the absorbance at 450 and 540 nm was measured using the Synergy H1 plate reader (BioTek Instruments).

### Western blot analysis

1.5 × 10^5^ Caco-2 cells/well (24-well plate) were treated with 0.1 mg/mL of DHA sample for 24 h. After incubation, the cells were stimulated with H_2_O_2_ (1.0 mM) or LPS (1 μg/mL) or vehicle for 24 h, then the cell culture media were collected in an ice-cold microcentrifuge tube and processed for the Griess assay and for cytokine quantifications. Meanwhile the cells were scraped in 30 µL ice-cold lysis buffer [RIPA buffer + inhibitor cocktail + 1:100 PMSF + 1:100 Na-orthovanadate] and transferred in an ice-cold microcentrifuge tube. After centrifugation at 13,300*g* for 15 min at 4 °C, the supernatant was recovered and transferred in a new ice-cold tube. Total proteins were quantified by the Bradford method and 50 μg of total proteins loaded on a pre-cast 7.5% sodium dodecyl sulphate-polyacrylamide (SDS-PAGE) gel at 130 V for 45 min. Subsequently, the gel was pre-equilibrated with 0.04% sodium dodecyl sulphate in H_2_O for 15 min at RT and transferred to a nitrocellulose membrane (mini nitrocellulose transfer packs), using a Trans-blot Turbo at 1.3 A, 25 V for 7 min. Target proteins, on milk or BSA blocked membrane, were detected by primary antibodies as follows: anti-iNos and anti-β-actin. Secondary antibodies conjugated with HRP and a chemiluminescent reagent were used to visualize target proteins and their signal was quantified using the Image Lab Software (Biorad). The internal control β-actin was used to normalize loading variations.

### Statistical analysis

Continuous variables were checked for normality using the Shapiro–Wilk Test. For normally distributed variables one-way ANOVA was performed followed by Tukey’s post-test for multiple comparisons among groups. For not-normally distributed variables Friedman test and Dunn’s test for multiple comparison were applied. For KIDMED score the Wilcoxon matched pairs signed rank test was performed.

All results were expressed as the mean ± standard deviation (SD) and p-values < 0.05 were considered statistically significant. Statistical analysis was performed using Graphpad Prism 9 (GraphPad Software. La Jolla. CA. USA).

As per the microbiota analysis, statistical evaluation of the alpha-diversity indices and of the taxonomic relative abundances was performed by a two-sample Wilcoxon test (Mann–Whitney test), whereas beta-diversity differences were assessed by a permutation test with pseudo F-ratios (“adonis” test) [50].

## Results

### Pediatric cohort characteristics

We initially recruited 18 children, among them 8 patients dropped out due to lack of interest in pursuing the study and the dietary intervention protocol. Ten children were included in the final analysis (mean age 8.85 ± 2.17; 7 females and 3 males). Among all the participants, 7 children were classified as obese (> + 2 SDS WHO growth charts) whilst 3 children were classified as severe obese (> + 3 SDS WHO growth chart. All the children at baseline showed a WHtR value greater than 0.5, apart from one participant. Baseline characteristics of the pediatric age group are shown in Table 1.

**Table 1:** Baseline characteristics of the pediatric age cohort expressed as mean ± SD

Baseline characteristics	Mean ± SD
Age (y)	8.85 ± 2.17
Weight (kg)	49.39 ± 13.02
Height (cm)	139.2 ± 18.5
BMI (kg/m^2^)	24.86 ± 3.70
BMI SDS WHO	2.76 ± 0.46
SBP (mmHg)	110.9 ± 0.67
DBP (mmHg)	67.7 ± 3.8
WC (cm)	75.9 ± 9.69
WC z score	1.64 ± 0.3
TSF (mm)	31.3 ± 7.51
TSF z score	2.65 ± 0.4
WHtR	0.545 ± 0.04
FM %	32.68 ± 6.48
FM (kg)	16.62 ± 7.35
FFM %	67.42 ± 6.51
FFM (kg)	32.67 ± 9.33
FM index	11.68 ± 4.43
FFM index	23.14 ± 4.30

*BMI* body mass index, *SDS* standard deviation score, *SBP* systolic blood pressure, *DBP* diastolic blood pressure, *WC* waist circumference, *TSF* tricipital skinfold thickness, *WHtR* waist-to-height-ratio, *FFM* fat free mass, *FM* fat mass

During the study period we observed a significant trend toward the increasing of weight and height (Table 2). The BMI value significantly increases at the end of study period compared to baseline, although no significant differences were found when evaluating BMI SDS across the study period. The body composition assessment shows a significant increase in FFM in kg at T2 compared to T1 (37.44 ± 13.3 at T2 vs 32.40 ± 9.4 at T1, p = 0.018), consistent with the pediatric patients’ growth. A marginally significant decrease in FFM% was observed at T1 in respect to baseline (63.70 ± 8.3 at T1 vs 67.42 ± 6.5 at T0, p = 0.049).

**Table 2:** Anthropometric parameters and body composition, expressed as mean ± SD, at different timepoints: T0 (baseline), T1 (4 months, after supplementation with DHA and dietary intervention), T2 (8 month after dietary intervention)

Variable	T0	T1	T2	*p*-value
Weight*	49.39 ± 15.6^a^	52.01 ± 17.0^a^	54.27 ± 18.9^b^	***0.006***
Height	139.2 ± 13.0^a^	142.1 ± 13.2^b^	143.2 ± 12.7^b^	** < *0.0001***
BMI*	24.86 ± 3.7^a^	25,15 ± 4.5^a,b^	25.70 ± 4.9^b^	***0.030***
BMI SDS (WHO)	2.76 ± 0.5	2.67 ± 0.7	2.67 ± 0.6	0.659
WC	75.90 ± 9.7	76.45 ± 10.8	79.0 ± 12.2	0.058
WC SDS	1.64 ± 0.3	1.54 ± 0.4	1.58 ± 0.4	0.514
TSF*	31.30 ± 7.5	32.75 ± 8.3	29.20 ± 8.0	0.444
TSF SDS	2.65 ± 0.4	2.71 ± 0.6	2.37 ± 0.5	0.226
WHtR	0.545 ± 0.04	0.537 ± 0.05	0.551 ± 0.06	0.273
FM %	32.68 ± 6.5	36.29 ± 8.3	34.50 ± 7.9	0.071
FM kg	16.62 ± 7.3	19.56 ± 9.6	20.07 ± 10.3	0.054
FFM%	67.42 ± 6.5^a^	63.70 ± 8.3^b^	65.59 ± 7.9^a,b^	**0.049**
FFM kg*	32.67 ± 9.3^a,b^	32.40 ± 9.4^a^	37.44 ± 13.3^b^	***0.018***
FM index	11.68 ± 4.4	13.49 ± 5.9	13.73 ± 6.4	0.092
FFM index*	23.14 ± 4.3	22.50 ± 4.4	25.80 ± 7.8	0.093

*BMI* body mass index, *SDS* standard deviation score, *WC* waist circumference, *TSF* tricipital skinfold thickness, *WHtR* waist-to-height-ratio, *FFM* fat free mass, *FM* fat mass

*For normally distributed variables one-way ANOVA was performed. For not normally distributed variables (*) Friedman Test was performed. Different superscript letters indicate statistically significant differences (p < 0.05)

Regarding hematological parameters no significant differences were observed, except for ERS, which showed a significant increase at T1 compared to baseline (34.60 ± 16.4 at T1 vs 24.30 ± 12.7 at T0, p = 0.03) (Table 3).

**Table 3:** Hematological parameters and blood pressure, expressed as mean ± SD, at different timepoints: T0 (baseline), T1 (4 months, after supplementation with DHA and dietary intervention), T2 (8 month after dietary intervention)

Variable	T0	T1	T2	*p*-value
Glycemia	87.60 ± 5.8	85.5 ± 5.3	88.1 ± 7.8	0.319
Insulin*	13.94 ± 6.7	17.07 ± 10.5	14.38 ± 7.4	0.897
HOMA index*	3.07 ± 1.7	3.64 ± 2.4	3.19 ± 1.9	0.974
QUICKI*	0.33 ± 0.03	0.32 ± 0.02	0.33 ± 0.02	0.730
VAI index*	1.23 ± 0.96	1.42 ± 1.3	1.31 ± 0.93	0.830
Mcauley index	7.27 ± 1.9	6.61 ± 1.6	7.17 ± 1.6	0.360
Glycated haemoglobin	34 ± 3.4	33.9 ± 3.1	33.9 ± 1.7	0.981
Cholesterol	158.4 ± 27.5	168.1 ± 28.3	159 ± 33.6	0.268
LDL-C	102.8 ± 22.1	110.5 ± 24.7	106.7 ± 29.6	0.285
HDL-C*	47.7 ± 12.2	48.3 ± 12.2	45.8 ± 13.4	0.186
Triglycerides*	78.9 ± 45.5	89.8 ± 50.7	75.4 ± 35.1	0.368
TyG index*	8.03 ± 0.49	8.17 ± 0.47	8.01 ± 0.48	0.710
TG/HDL-C index*	1.80 ± 1.2	2.07 ± 1.5	1.86 ± 1.1	0.830
AST	26.8 ± 6.4	28.7 ± 6.9	27 ± 6.9	0.514
ALT*	22 ± 9.3	20.4 ± 10.1	16.2 ± 3.8	0.094
GGT*	14.3 ± 4.8	15.8 ± 5.9	15.7 ± 7.0	0.494
ESR*	24.30 ± 12.7^a^	34.60 ± 16.4^b^	31.10 ± 19.7^a,b^	***0.003***
SBP	110.9 ± 6.9	114.5 ± 9.5	106.8 ± 11.2	0.165
DBP	67.70 ± 3.8	65.50 ± 6.8	64.90 ± 6.4	0.436

*HOMA-IR* homeostasis model assessment of insulin resistance; *QUICKI* quantitative insulin-sensitivity check index, *TyG* triglyceride–glucose index, *VAI index* visceral adiposity index, *LDL-C* low-density lipoproteins cholesterol, *HDL-C* high-density lipoproteins cholesterol, *AST* aspartate aminotransaminase, *ALT* alanine aminotransferase, *GGT* gamma-glutamyl transferase, *ESR* erythrocyte sedimentation rate, *SBP* systolic blood pressure, *DBP* diastolic blood pressure

*For normally distributed variables one-way ANOVA was performed. For not normally distributed variables (*) Friedman Test was performed. Different superscript letters indicate statistically significant differences (p < 0.05)

Dietary intakes for each timepoint (T0, T1, T2) are listed in Table S1. No significant differences were observed for any of the nutritional intakes. In line with this, the average score of the KIDMED questionnaire all along the study period remained below the score of 8 points, which is defined as the threshold for good adherence to the Mediterranean diet. We observed a slight increase of the mean score at T1 although without significance (6.6 ± 2.4 at T0; 7.9 ± 1.8 at T1; p = 0.10). No significant changes were observed at T2 (mean score 7.3 ± 2.1) compared to both baseline and T1.

### Analysis of fecal microbiota composition

**Fig. 1:**
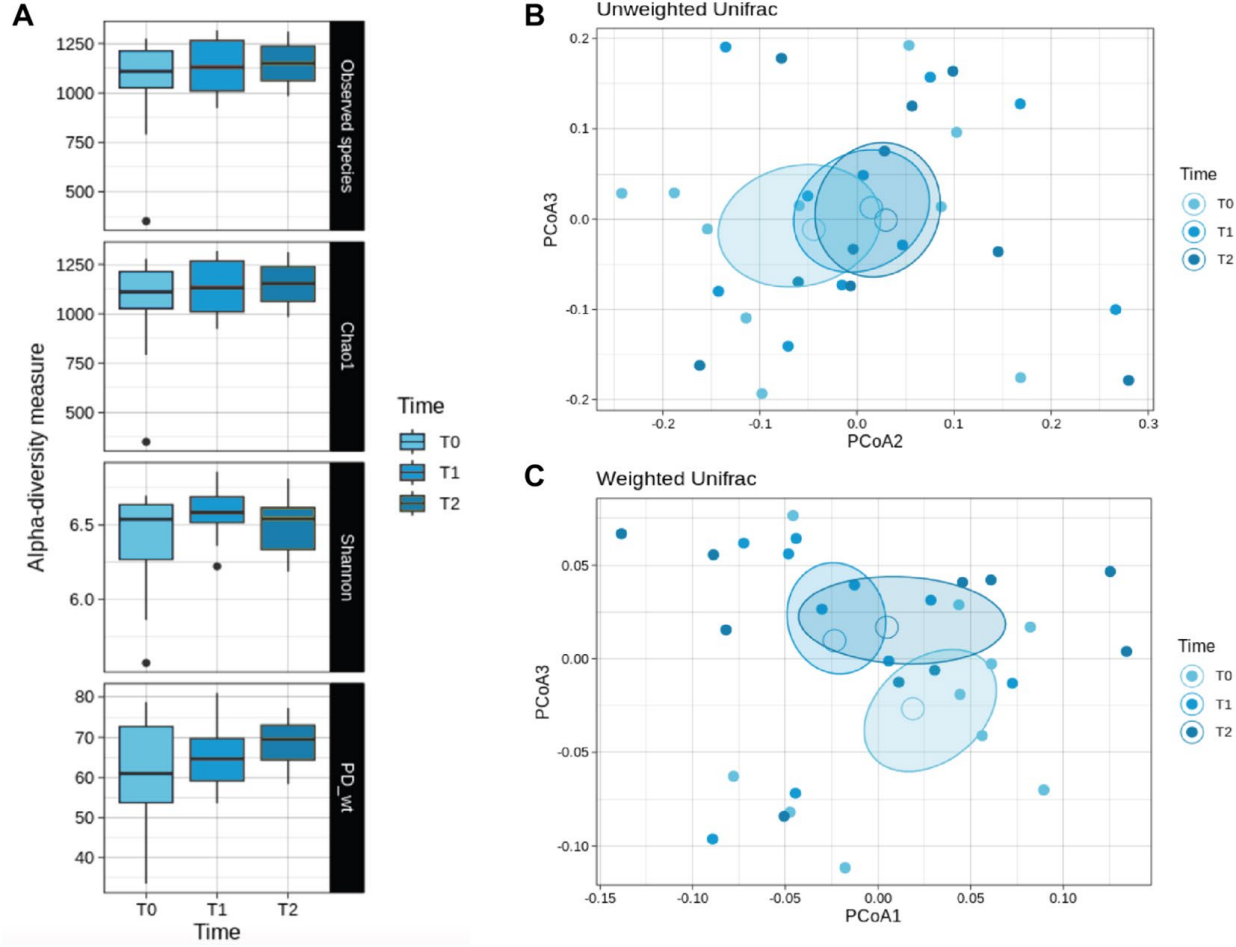
Gut microbial diversity of obese children before and after DHA supplementation. **A** Boxplots showing the alpha-diversity measures within 4 metrics (observed species, Chao1, Shannon, PD whole tree). No statistical differences were found. Beta-diversity was observed through the Principal Coordinate Analysis of the unweighted (**B**) and weighted (**C**) Unifrac matrix of dissimilarity. The first and second principal coordinates are reported for both measures. Comparisons not significant

The gut microbiota was characterized by next-generation sequencing using V3–V4 hypervariable 16S rRNA genomic region. We evaluated possible changes in the microbiota composition of children with obesity before (T0) and after DHA supplementation (T1), and whether these changes might last after DHA supplementation discontinuation (T2). We did not observe significant differences in the alpha-diversity analysis, for all metrics (see also Table S2), after DHA supplementation (Fig. 1, panel A). Similarly, the Principal Coordinate Analysis of the unweighted (Fig. 1, panel B) and weighted (Fig. 1, panel C) Unifrac matrix of dissimilarity did not show significant modifications in the beta-diversity.

**Fig. 2:**
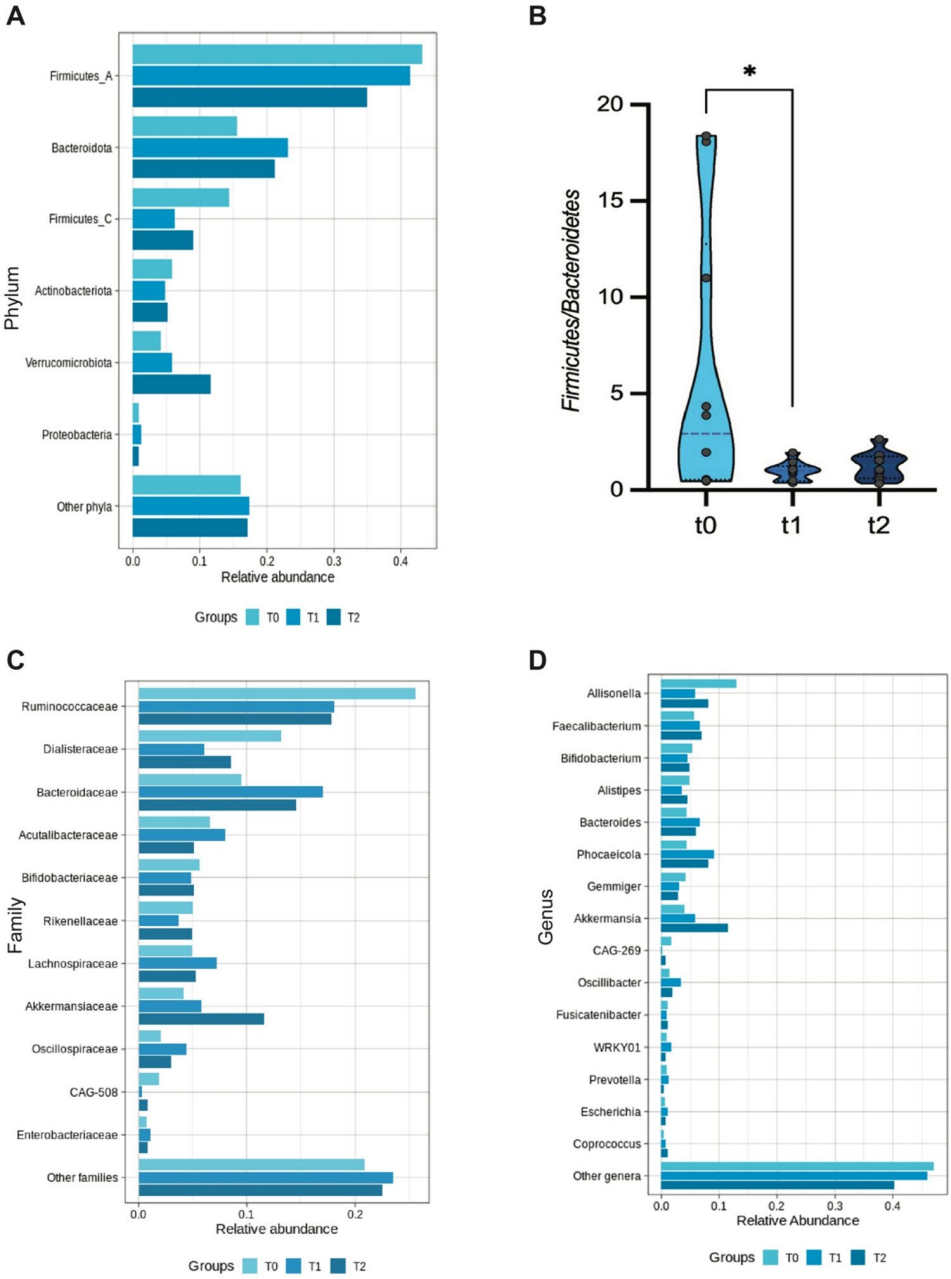
Taxonomy analysis. **A** Bacterial composition was characterized at the phylum (**A**), family (**C**), and genus (**D**) phylogenetic levels. Bacterial groups with a relative abundance lower than 1% were clustered into the “Other” group. Panel **B**, Firmicutes/Bacteroidetes ratio at t0, after DHA supplementation (t1), and after DHA discontinuation (t2). The ratio is significantly lower after DHA treatment (Wilcoxon test, p = 0.0137)

We then analyzed DHA-related differences in the relative abundances at different taxonomic levels (Fig. 2, Table S3).

Firmicutes were found the most abundant phylum, with a cumulative relative abundance (mean ± SD) of 58% ± 28.3, followed by bacteroidetes (15.6 ± 12.7), actinobacteria (5.8 ± 4.1), and verrucomicrobia 4.1 ± 6.0 (Fig. 2, panel A). F/B ratio was elevated and with large interindividual variation. Four-months DHA supplementation (T1) decreased the Firmicutes relative abundance and increased the bacteroidetes one, reducing and improving the F/B ratio (Fig. 2, panel B). This ratio was conserved after the DHA discontinuation. Besides, verrucomicrobia phylum progressively increased from T0 to T2, even though without statistical significance due to the high interindividual variation. At lower taxonomic level, DHA supplementation drove a depletion in Ruminococcaceae and *Dialisteraceae* and enrichment in *Bacteroidaceae*, *Oscillospiraceae*, and *Akkermansiaceae* (Fig. 2, panel C).

At genus level, *Allisonella* was the most decreased by DHA supplementation, whereas *Akkermansia* and *Phocaeicola* were the most enriched (Fig. 2, panel D).

### Evaluation of the DHA in vitro radical scavenging activity by DPPH and FRAP assays.

**Fig. 3:**
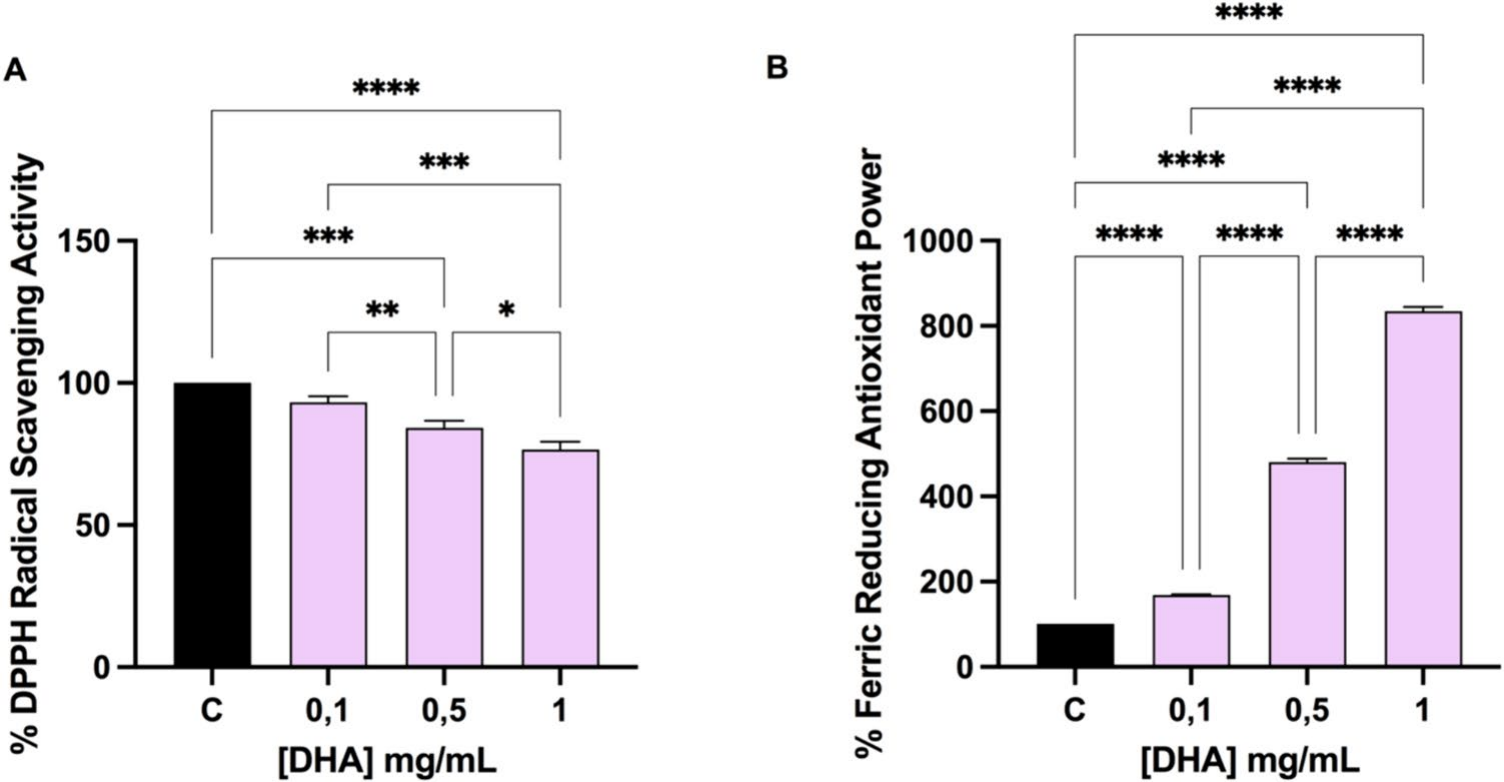
In vitro radical scavenging activity of DHA by DPPH (**A**) and FRAP (**B**) assays, respectively. Data represent the mean ± SD of four determinations performed in duplicate. All the data sets have been analyzed by One-way ANOVA followed by Tukey’s post-hoc test. *p < 0.1, **p < 0.01, ***p < 0.001, ****p < 0.0001

To assess the antioxidant properties of DHA, the DPPH and FRAP assays were performed. As reported in Fig. 1A, results suggest that DHA scavenged the DPPH radical by 6.8 ± 2.1% at 0.1 mg/mL, by 15.81 ± 2.5% at 0.5 mg/mL and 23.4.8 ± 2.7% at 1.0 mg/mL. Moreover, DHA increased the FRAP by 69.08 ± 1.5%, 380.8 ± 8.2% and 734.4 ± 10.5% at 0.1, 0.5 and 1.0 mg/mL, respectively (Fig. 3, panel B).

### Evaluation of Caco-2 cells viability

Based on the previously obtained results, the cellular evaluations of DHA antioxidant properties were carried out. Before proceeding to experiments on Caco-2 cells, it was necessary to perform the MTT experiments for verifying that the compound did not impair cellular vitality. After testing several DHA concentrations ranging from 0.1 to 10 mg/ml, no significant changes on Caco-2 viability were found compared to control (see Figure S1).

### DHA decrease the H_2_O_2_-induced ROS and lipid peroxidation levels in human intestinal Caco-2 cells

**Fig. 4:**
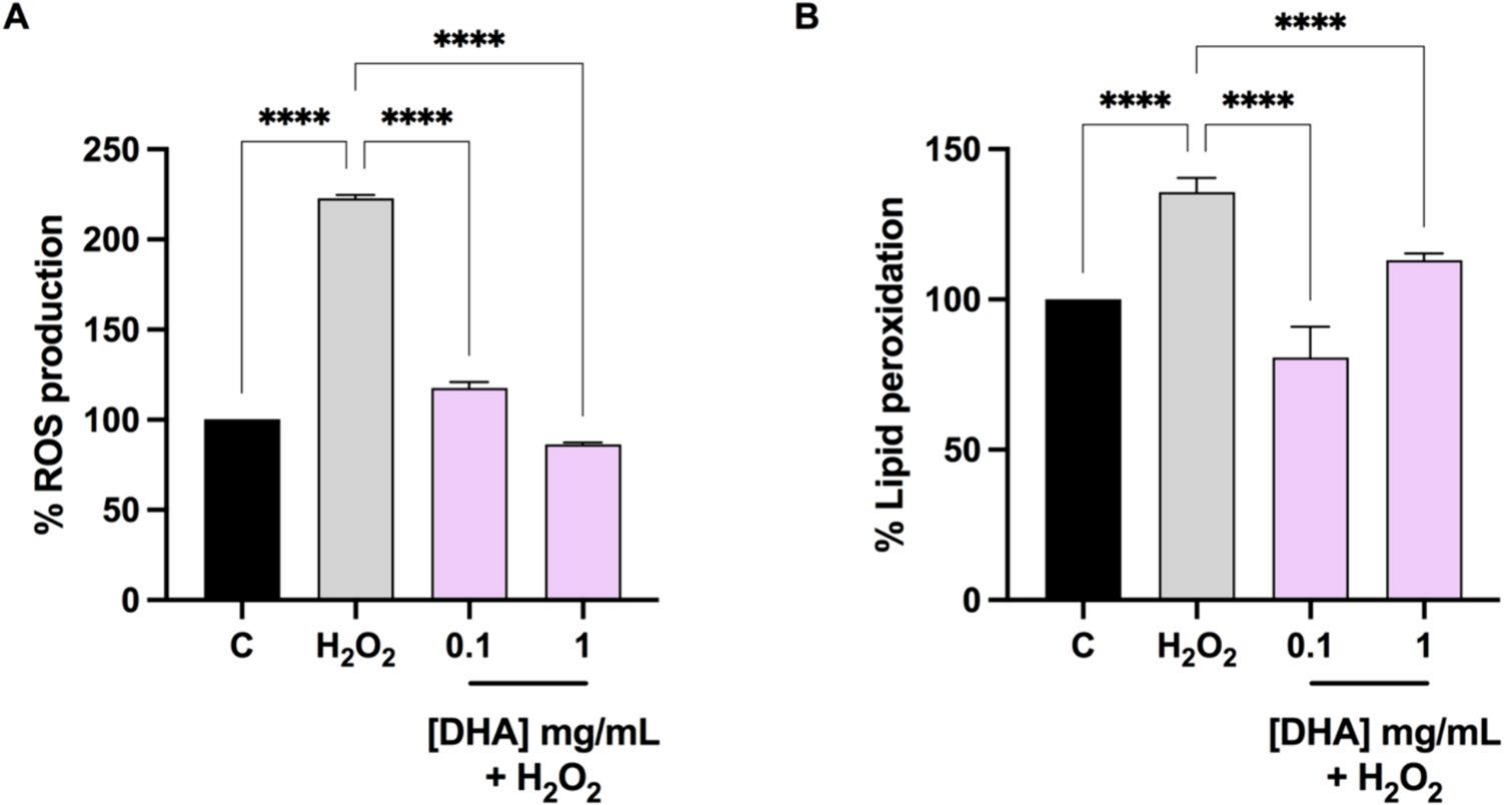
**A** Modulation of intracellular ROS production (**A**) and lipid peroxidation levels by means of MDA assay (**B**), induced by H_2_O_2_ and by H_2_0_2_ with DHA pretreatment. Data represent the mean ± SD of six independent experiments performed in triplicate. All data sets were analyzed by One-way ANOVA followed by Tukey’s post-hoc test. **C** control sample; ns: not significant; *p < 0.05, **p < 0.01, ****p < 0.0001. *C* control, *ROS* reactive oxygen species, *DHA* docosahexaenoic acid

To evaluate the ability of DHA to vary ROS overproduction induced by H_2_O_2_, cellular experiments were carried out. Our findings clearly demonstrated that Caco-2 cells treated with H_2_O_2_ alone showed an increase of ROS levels up to 222.7 ± 1.9%, versus the control cells. On the contrary, the presence of DHA reduced the intracellular H_2_O_2_-induced ROS levels up to 117.5 ± 3.4% and 86.3 ± 1.0% at 0.1 and 1 mg/mL respectively (Fig. 4, panel A).

In addition, for evaluating the capacity of DHA to modulate the H_2_O_2_-induced lipid peroxidation in human intestinal Caco-2 cells, the MDA assay was performed. In agreement with the enhancement of ROS production after the same treatment with H_2_O_2_, a noticeable increase of the intracellular lipid peroxidation was detected up to 135.7 ± 4.7% versus the control cells. In addition, our results showed that the pre-treatment with DHA resulted in a decrease in MDA levels up to 80.32 ± 10.3%, and 113.7 ± 3.2%, at 0.1 and 1 mg/mL respectively (Fig. 4, panel B).

### DHA modulate the H_2_O_2_ and LPS-induced NO level production via the iNOS protein level modulation in Caco-2 cells

**Fig. 5:**
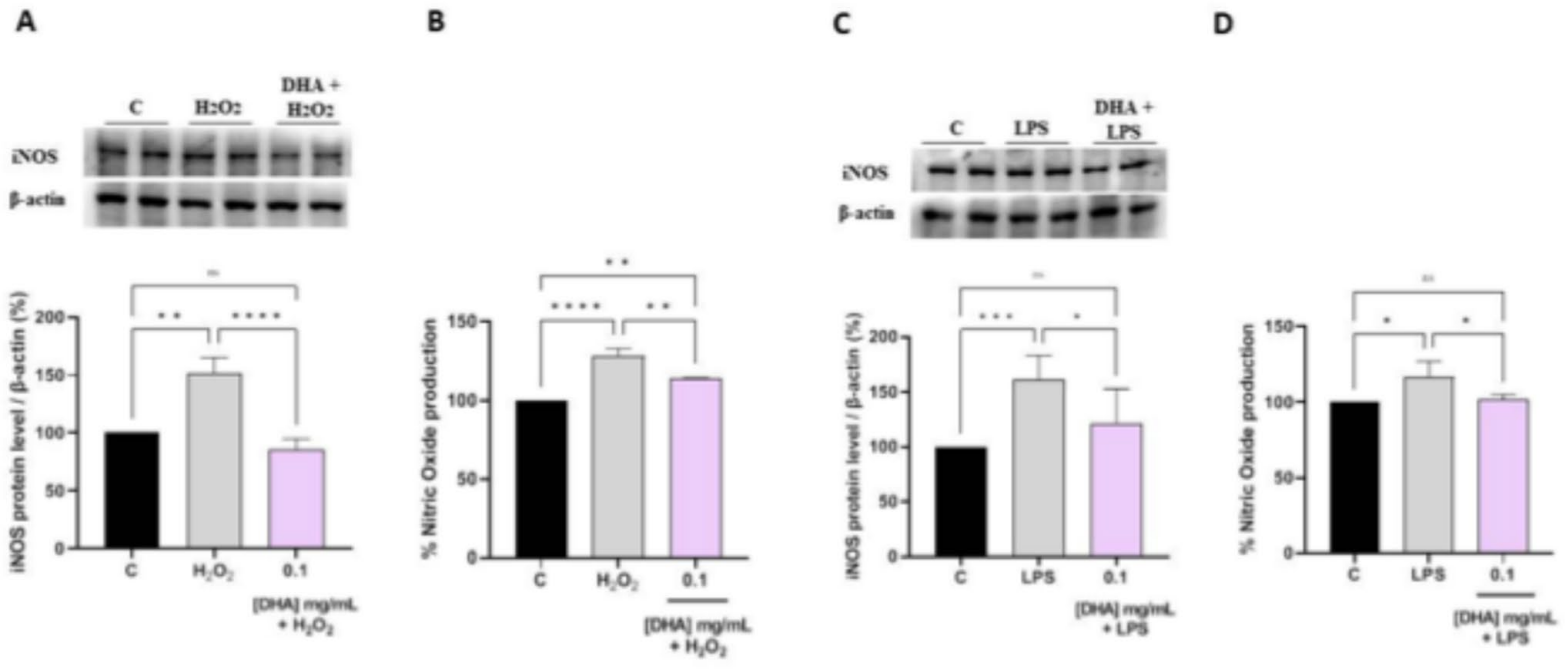
Effect of DHA on the H_2_O_2_ (1 mM) and LPS-induced inducible nitric oxide synthase (iNOS) protein levels (**A**–**C**) and NO production (**B**–**D**) in human intestinal Caco-2 cells. The data points represent the averages ± SD of four independent experiments in duplicate. All data sets were analyzed by One-way ANOVA followed by Tukey’s post-hoc test. **C** Control sample; ns: not significant; *p < 0.05, **p < 0.01, ****p < 0.0001. *C* control, *iNOS* inducible-nitric oxide synthase, *DHA* docosahexaenoic acid, *LPS* lipopolysaccharides

The effects of DHA on the production of NO levels were evaluated on human intestinal Caco-2 cells after oxidative stress induction. Notably, H_2_O_2_ (1 mM) treatment induced an oxidative stress that led to an increase of intracellular NO levels up to 128.2 ± 4.8%. Pre-treatment with DHA reduced the H_2_O_2_-induced NO overproduction up to 114.2 ± 0.9%, at 0.1 mg/mL (Fig. 5, panel B).

In parallel, the effects of DHA on iNOS protein levels were assessed after oxidative stress induction by western blot experiments, in which the iNOS protein band at 130 kDa was detected and quantified. After H_2_O_2_ treatment (1 mM), the iNOS protein increased up to 151.1 ± 14.4% in Caco-2 cells (Fig. 5, panel A). The pre-treatment with 0.1 mg/mL of DHA reduced the iNOS levels up to 85.3 ± 9.5% (Fig. 5 panel A). Similarly, also the LPS stimulation induced an inflammatory state in intestinal Caco-2 cells, that lead to a significant increase in the iNOS and NO level productions up to 161.5 ± 21.8% (p ≤ 0.001) (Fig. 5, panel C) and 117.0 ± 10.2% (Fig. 5, panel D), respectively. The treatment with DHA showed a significant reduction in iNOS and NO production, whose values were close to the baseline values. Specifically, DHA reduced the iNOS protein levels up to 121.4 ± 31.8% (p < 0.05) (Fig. 5, panel C) and NO production up to 101.7 ± 3.5% (p < 0.05) at 0.1 mg/mL (Fig. 5, panel D).

### DHA modulates the LPS-induced cytokine production in Caco-2 cells

**Fig. 6:**
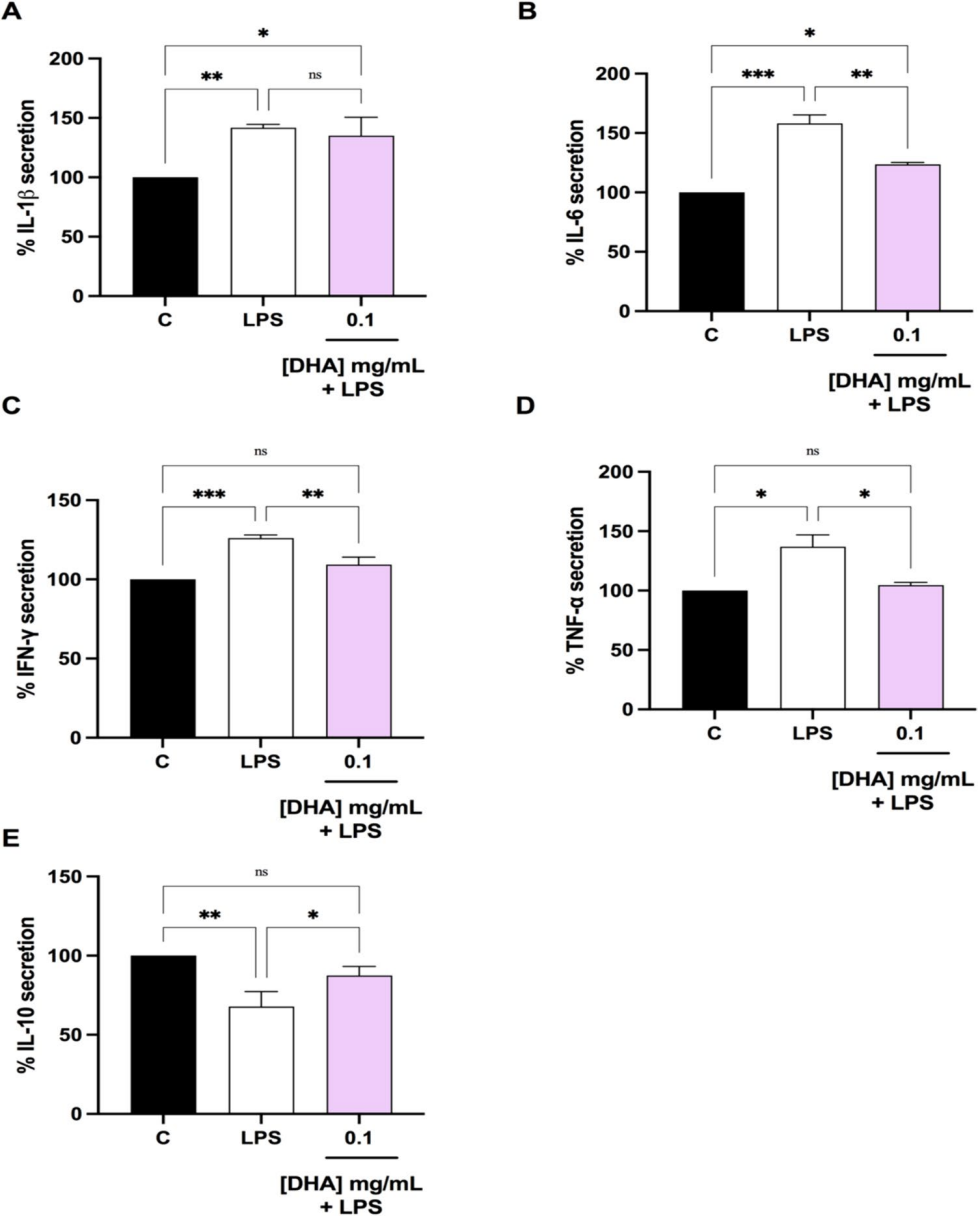
Effect of DHA on the LPS-induced pro-inflammatory (**A**–**D**) and anti-inflammatory (**E**) cytokines in human intestinal Caco-2 cells. The data points represent the averages ± SD of four independent experiments in duplicate. All data sets were analyzed by One-way ANOVA followed by Tukey’s post-hoc test. *C* control sample, *ns* not significant; *p < 0.05, **p < 0.01, ****p < 0.0001. *C* control, *DHA* docosahexaenoic acid, *LPS* lipopolysaccharides, *IFN-γ* interferon gamma, *IL* interleukin, *TNF-α* tumor necrosis factor alpha

To verify its anti-inflammatory and immunomodulator properties, the effect of DHA treatment on the production of pro-inflammatory (IL-1β, IL-6, IFN-γ, and TNF- α) and anti-inflammatory (IL10) cytokines was determined in LPS-stimulated Caco-2 cell culture supernatants. As shown in Fig. 6, the LPS stimulation increased the production of the pro-inflammatory cytokines, and reduced the IL-10 production, compared with LPS-unstimulated cells (untreated control, C). More in details, LPS induced the IL-1β, IL-6, IFN-γ, and TNF- α secretion up to 141.7 ± 2.9% (p < 0.01), 158.0 ± 7.3% (p < 0.001), 126.1 ± 1.9% (p < 0.001), respectively, and reduced the IL-10 production up to 67.9 ± 9.5% (p < 0.01) (Fig. 6A–E). The DHA pretreatment at 0.1 mg/mL significantly reduced the LPS-induced IL-6 production up to 123.6 ± 1.7% (Fig. 6, panel B, p < 0.01) and restored the LPS-induced IFN-γ and TNF-α secretion up to 109.3 ± 4.7% and 104 ± 2.3% (Fig. 6 panels C and D), respectively, whereas no significant changes were observed with DHA for LPS-induced IL-1β secretion (Fig. 6, panel A). In addition, DHA significantly increased IL-10 levels up to 87.4 ± 5.8% (Fig. 6, panel E), which were restored to a level not significantly different compared to the control.

## Discussion

In the present study, despite the small cohort, we corroborated previous data [21] showing that childhood obesity is characterized by an altered gut microbiota with increased levels of firmicutes and depleted levels of bacteroidetes. As described in Riva et al. BMI z-score positively correlated with the abundance of firmicutes, driving the increase in the F/B ratio. The F/B ratio is still a parameter highly discussed in papers investigating gut dysbiosis and obesity, as it could reflect the fermentative capabilities of gut microbiota to extract calories from nutrients and to produce specific metabolites [51]. Regarding dietary intervention, no significant changes were reported for nutritional intakes, but only a slight increase in average KIDMED score, a measure of mediterranean diet adherence, was observed. The improvement in diet quality, although without significance, might have synergistically foster the gut microbiota changes. Nevertheless, as already reported in the EVASYON study, gut microbiota might be highly resilient to diet-induced changes and modifications in microbial ecosystem might occur only after a long environmental pressure [18, 19, 52]. For instance, Ruiz and coworkers reported major changes in the gut microbiota structure and improvement at functional level (expressed as α-glucosidase and β-galactosidase activities), only after 1-year calorie restriction dietary intervention in adolescents with obesity [18]. In the present study, according to the nutritional and behavioral survey, none of the enrolled child implement the lifestyle following physician’s suggestions. Hence, the observed effects on the gut microbial community seem to be ascribable to the DHA supplementation itself.

Nevertheless, we observed a high interindividual variation either in the basal gut microbial community (T0) or in response to DHA intake (T1). Despite not observing statistically significant variation in the richness and evenness of gut microbiota (alpha-diversity), at community level (beta-diversity) we detect a shift in unweighted UniFrac distance with respect to T0, with T2 (after DHA discontinuation) almost overlapping T1. Indeed, when analyzing the taxa relative abundances, those varying after DHA intake did not return to the basal level after the wash out period.

In particular, we observed an increase in the relative abundance of the genus *Akkermansia*, recently enlisted in the next-generation probiotic with several animal studies demonstrating its role in preventing/modulating the metabolic syndrome-related features [53, 54]. *Akkermansia* expansion seems to be triggered by DHA and to continue after discontinuation, as its enrichment is even more pronounced at T2. On the contrary, *Bacteroidaceae* strongly increases during the DHA intake and slightly decreases after discontinuation. This trend is conserved at genus level, i.e., for *Phoaecicola* spp (formerly *Bacteroides* spp.) *P. vulgatus* and *P. dorei* have been reported as key microbial species that are depleted in individuals with obesity [55]. An increase in their relative abundance has been demonstrated in animal models to ameliorate the serum lipid profile and systemic inflammation [56], and to reduce the plasmatic levels of branched-chain amino acids and α-ketoacids, hallmarks of obesity [57].

Among the decreased taxa, we found *Allisonella*, that together with other genera such as *Slackia*, *Ruminococus_2*, *Megasphaera, Escherichia/Shigella*, and *Prevotella*, has been recently suggested as biomarker species for prediabetes or type-2 diabetes [58]. This reduction is meaningful as *Allisonella* has been demonstrated to promote inflammation by producing histamine and other inflammatory molecules [59].

The ability to counteract inflammation through modulation of gut microbiota, matches also with the antioxidant and anti-inflammatory effect of DHA on human colonocytes. DHA possesses strong antioxidant properties, primarily attributed to its molecular structure, which includes multiple double bonds. These double bonds allow DHA to readily donate electrons to neutralize free radicals, thereby preventing oxidative damage to cellular components such as lipids, proteins, and DNA. Indeed, DHA displayed the direct ability to scavenge the DPPH radicals, whereas it shows a dose-dependent FRAP activity. We corroborated previous data, showing that DHA is safe for intestinal cells at several concentrations (ranging from 0.1 to 10 mg/ml). At cellular level, we have decided to use H_2_O_2_ as stimulus for inducing the oxidative stress [60, 61] and LPS as stimulus for the development of inflammatory condition [62]. Results indicated that H_2_O_2_ clearly enhances the ROS and lipid peroxidation level in colonocytes, and DHA clearly restored toward physiological condition, the H_2_O_2_ induced ROS and the lipid peroxidation, respectively (Fig. 4).

These results agree with the DHA ability to modulate the intracellular NO levels via iNOS pathway modulation (Fig. 5). In fact, we demonstrated that the pre-treatment of Caco-2 cells with DHA reduced the H_2_O_2_-induced iNOS protein level, bringing their levels close to basal conditions. Due to the cross link between oxidative stress and inflammatory condition [63], we have clearly demonstrated that beside iNOS pathway modulation, DHA exert anti-inflammatory effect through the reduction of the LPS induced pro-inflammatory cytokines (IL-6, IFN-γ, and TNF- α), and improving the LPS reduced anti-inflammatory cytokines (IL-10), while no effects were observed on IL-1β secretion (Fig. 6). In this context, one key mechanism might involve its conversion into specialized pro-resolving lipid mediators (SPMs), such as resolvins, protectins, and maresins. In colonocytes, these SPMs actively might resolve inflammation by dampening the production of pro-inflammatory cytokines induced by LPS. Overall, this study clearly provides piece of information that DHA exerts antioxidant and anti-inflammatory effects in Caco-2 cells through multiple mechanisms, including scavenging free radicals and signaling pathways modulation. These findings highlight the potential therapeutic benefits of DHA supplementation in mitigating inflammation and oxidative stress-related pathologies.

The lack of a control group of children with obesity on free diet is a limitation of the present study. Indeed, the recruitment of such a control group could raise ethical issues since the absence of intervention would not comply with the recommendation for the treatment of childhood obesity of the Italian Society of Pediatrics [33, 34]. Among the limitations of the present study, we need to acknowledge the high rate of dropout of children who did not complete the pilot study. Clinical studies with high statistical power are needed to quantify the extent to which DHA supplementation affects the gut microbiota in children and adolescents with obesity. At the pre-clinical level, studies at a higher level of complexity are required to characterize the anti-inflammatory and gut microbiota-modulating effect of DHA. DHA activity on ex-vivo model of the human intestinal microbial ecosystem has not yet been studied. Some studies on mice model are available [5–9], although this topic is still in its infancy.

In conclusion, this is the first study combining the health effects of DHA at clinical and pre-clinical level. DHA supplementation appears to improve gut dysbiosis of children with obesity, which seems to persist even after discontinuation. This potential ability to modulate gut microbiota is also compatible with the anti-inflammatory effects at pre-clinical level of DHA on human colonocytes.

## Supplementary Information

Below is the link to the electronic supplementary material.

Supplementary file1 (DOCX 85 KB)

Supplementary file2 (DOCX 72 KB)

Supplementary file3 (XLSX 10 KB)

Supplementary file4 (XLSX 13 KB)

## Data Availability

All pre-clinical data supporting the findings of this study are available within the paper and its Supplementary Information. The clinical and patient’s data that support the findings of this study are not openly available due to reasons of sensitivity and are available from the corresponding author upon reasonable request.

## Abbreviations

AA: Arachidonic acid
ALA: α-Linolenic acid
ALT: Alanine aminotransferase
AST: Aspartate aminotransaminase
ASV: Amplicon sequence variant
BHT: Butylated hydroxytoluene
BMI: Body mass index
BSA: Bovine serum albumin
CHO: Carbohydrates
CRP: C-reactive protein
DBP: Diastolic blood pressure
DHA: Docosahexaenoic acid
DMEM: Dulbecco’s modified Eagle’s medium
DMSO: Dimethyl sulfoxide
DNA: Deoxyribonucleic acid
DPPH: 1,1-Diphenyl-2-picrylhydrazyl
DPPH: 1,1-Diphenyl-2-picrylhydrazyl
EPA: Eicosapentaenoic acid
ESR: Erythrocyte sedimentation rate
F/B: Fimicutes/Bacteroidetes
FBS: Fetal bovine serum
FFM: Fat free mass
FFMI: Fat Free Mass Index
FM: Fat mass
FMI: Fat Mass Index
FRAP: Ferric reducing ability of plasma
GGT: Gamma-glutamyl transferase
GI: Glycemic index
GTDB: The Genome Taxonomy Database
HDL-C: High-density lipoproteins cholesterol
HOMA-IR: Homeostasis model assessment of Insulin resistance
HRP: Horseradish peroxidase
IFN-γ: Interferon gamma
IL: Interleukin
iNOS: Inducible-nitric oxide synthase (iNOS)
LA: Linoleic acid
LDL-C: Low-density lipoproteins cholesterol
LPS: Lipopolysaccharides
MCAi: McAuley index
MDA: Malondialdehyde
MTT: 3-(4,5-Dimethylthiazol-2-yl)-2,5-diphenyltetrazolium bromide
MUFA: Monounsaturated fatty acids
NO: Nitric oxide
PBS: Phosphate buffered saline
PMSF: Phenylmethanesulfonyl fluoride
PUFA: Polyunsaturated fatty acid
QUICKI: Quantitative Insulin-Sensitivity Check Index
RDP: Ribosomal Database Project
RIPA: Radioimmunoprecipitation assay
RNA: Ribonucleic acid
ROS: Reactive oxygen species
RT: Room temperature
SBP: Systolic blood pressure
SCFA: Short‐chain fatty acids
SD: Standard deviation
SDS: Standard deviation score
SDS-PAGE: Sodium dodecyl sulphate-polyacrylamide
SFA: Saturated fatty acids
SPM: Specialized pro-resolving mediators
TBA: Thiobarbituric acid
TG: Triglycerides
TNFα: Tumor necrosis factor alpha
TPTZ: 2,4,6-Tris(2-pyridyl)-s-triazine
TSF: Tricipital skinfold thickness
TyG: Triglyceride–Glucose index
VAI: Visceral Adiposity index
WC: Waist circumferences
WHO: World Health Organization
WHtR: Waist-to-height-ratio
