# Supplementary material for: Effect of docosahexaenoic acid as an anti-inflammatory for Caco-2 cells and modulating agent for gut microbiota in children with obesity (the DAMOCLE study)
Source: J Endocrinol Invest. 2024 Aug 26;48(2):465–81. doi: 10.1007/s40618-024-02444-w (PMC11785711; doi:10.1007/s40618-024-02444-w)
Supplement: Supplementary file 1 — Supplementary file1 (DOCX 85 KB) [file 40618_2024_2444_MOESM1_ESM.docx]

| ***Variable*** | ***T0*** | ***T1*** | ***T2*** | ***p-value*** |
| --- | --- | --- | --- | --- |
| Energy (kcal/day) | 1491 ± 275 | 1505 ± 252 | 1427 ± 232 | 0.750 |
| CHO (%En) | 51.3 ± 5,0 | 48.4 ± 7.1 | 48.5 ± 5.0 | 0.205 |
| Sugars (%En) | 15.6 ± 4.9 | 13.2 ± 3.8 | 12.3 ± 3.2 | 0.079 |
| Glycaemic Index | 56.0 ± 4.4 | 55.2 ± 6.0 | 55.4 ± 6.3 | 0.933 |
| Fibers (g/day) | 14.0 ± 2.2 | 12.4 ± 4.0 | 14.6 ± 3.2 | 0.281 |
| Lipids (%En)* | 34.4 ± 5.2 | 33.5 ± 3.6 | 34.1 ± 2.9 | 0.973 |
| SFA (%En) | 9.9 ± 2.0 | 8.7 ± 1.5 | 10.6 ± 2.5 | 0.161 |
| MUFA (%En) | 12.4 ± 3.4 | 12.2 ± 3.1 | 14.6 ± 1.8 | 0.168 |
| PUFA (%En)* | 3.02 ± 1.3 | 3.31 ± 1.2 | 3.70 ± 0.7 | 0.135 |
| Proteins (g/day) | 53.46 ± 9.7 | 63.43 ± 13.9 | 60.19 ± 8.3 | 0.109 |
| Proteins (g/kg/day) | 1.18 ± 0.41 | 1.31 ± 0.44 | 1.21 ± 0.36 | 0.389 |
| For normally distributed variables one-way ANOVA was performed. For not normally distributed variables (*) Friedman Test was performed. | | | | |

Table S1, Dietary intakes at different timepoints (mean±SD): T0 (baseline), T1 (4 months, after supplementation with DHA and dietary intervention), T2 (8 month after dietary intervention)


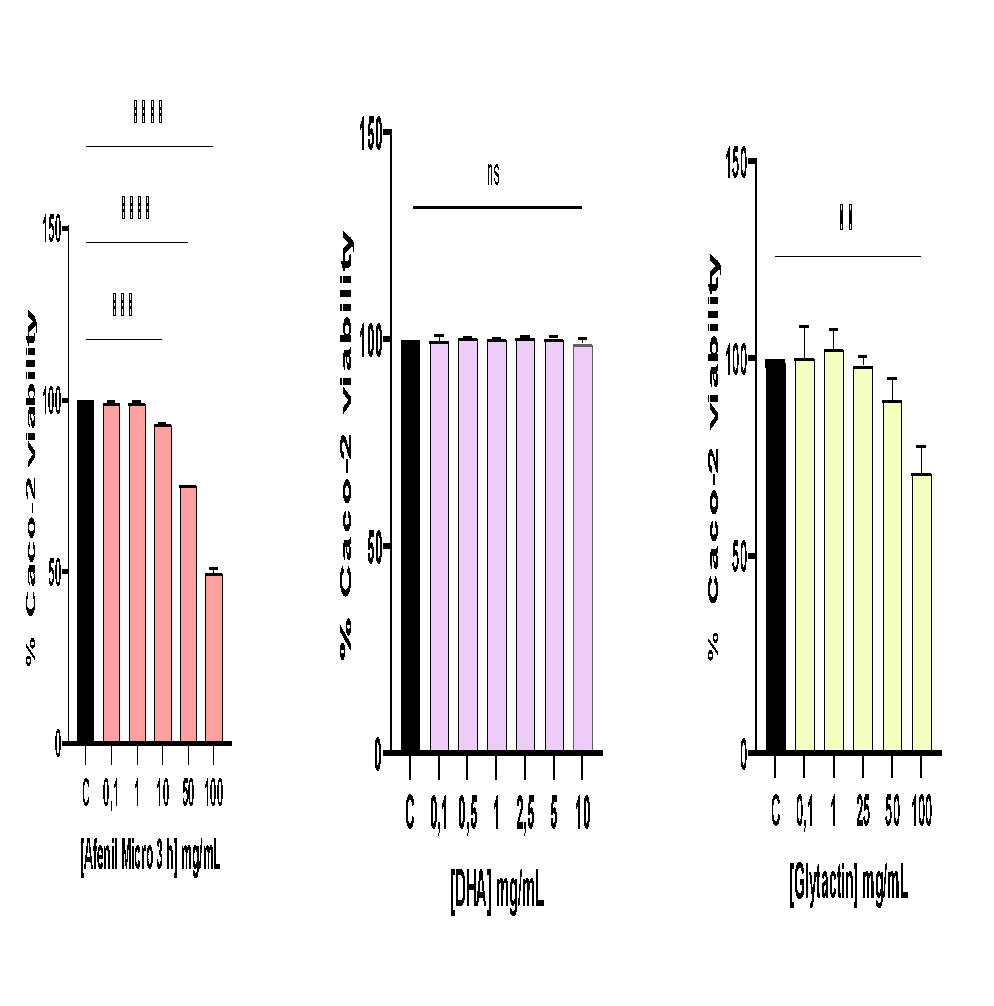


Figure S1, Effect of DHA on Caco-2 cells ‘viability by MTT experiments. (ns: not significant)
