# Supplementary material for: Effect of docosahexaenoic acid as an anti-inflammatory for Caco-2 cells and modulating agent for gut microbiota in children with obesity (the DAMOCLE study)
Source: J Endocrinol Invest. 2024 Aug 26;48(2):465–81. doi: 10.1007/s40618-024-02444-w (PMC11785711; doi:10.1007/s40618-024-02444-w)
Supplement: Supplementary file 2 — Supplementary file2 (DOCX 72 KB) [file 40618_2024_2444_MOESM2_ESM.docx]

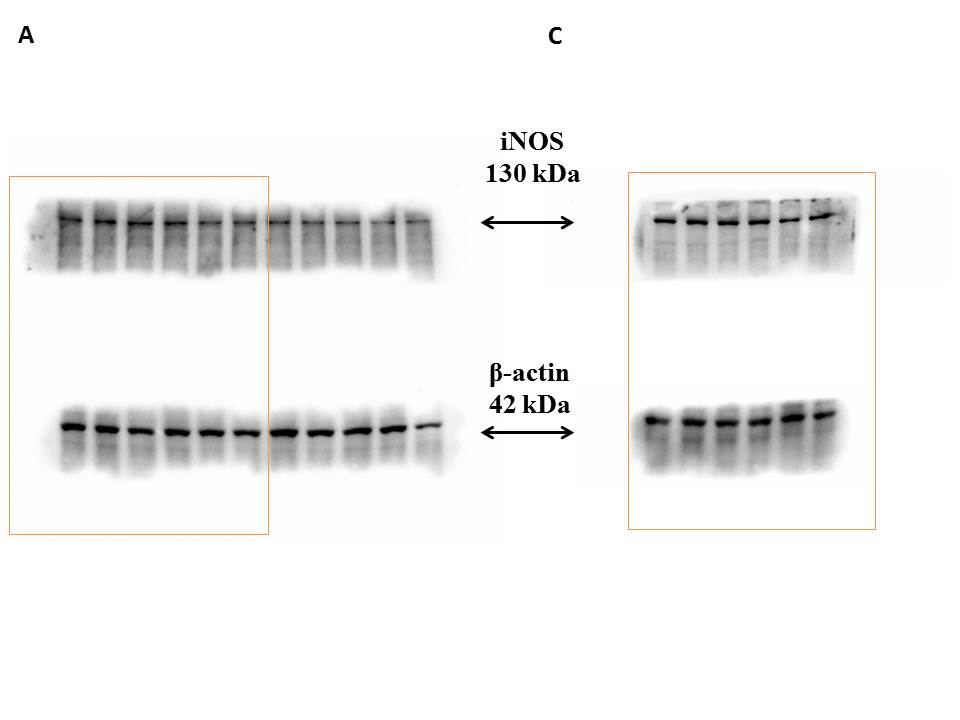


Figure S2. Collection of all Western blot analysis, uncropped image (see manuscript Figure 5, panel A and C). Panel A image was cut off as it had other samples loaded on the gel, not to be considered for the present study (the ones outside the yellow box).


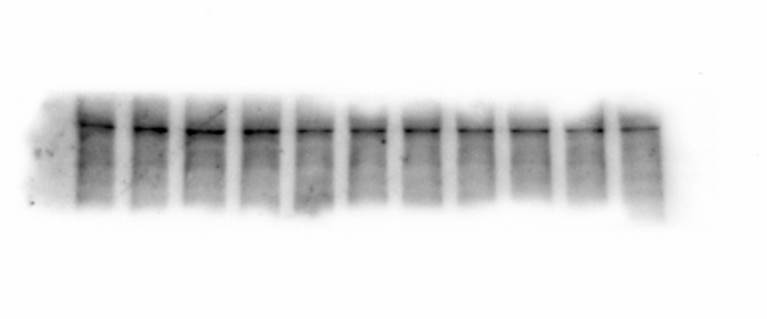


Figure S3 Western blot analysis, uncropped image, Figure 5 Panel A (iNOS protein levels). Inducible nitric oxide synthase (iNOS) protein levels production in human intestinal Caco-2 cells stimulated with H_2_O_2_ (1 mM) and DHA (only first 6 to be considered for the present study)


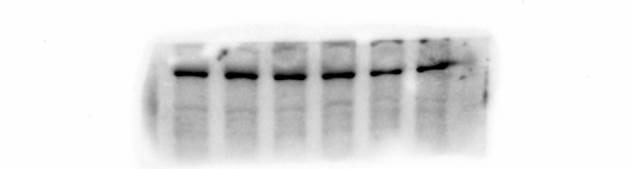


FigureS4. Western blot analysis, uncropped image, Figure 5 Panel C (iNOS protein levels). Inducible nitric oxide synthase (iNOS) protein levels production in human intestinal Caco-2 cells stimulated with LPS and DHA.


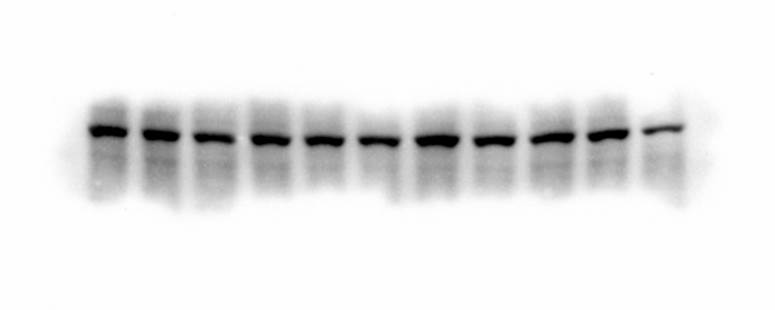


FigureS5. Western blot analysis, uncropped image, Figure 5 Panel A (β-actin levels). β-actin levels production in human intestinal Caco-2 cells stimulated with H_2_O_2_ (1 mM) and DHA (only first 6 to be considered for the present study)


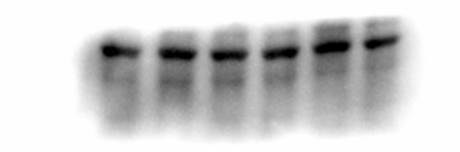


FigureS6. Western blot analysis, uncropped image, Figure 5 Panel C (β-actin levels). β-actin levels production in human intestinal Caco-2 cells stimulated with LPS and DHA

C
